# A multi-feature hybrid classification data mining technique for human-emotion

**DOI:** 10.1186/s13040-021-00254-x

**Published:** 2021-03-29

**Authors:** Y. Wang, Y. M. Chu, A. Thaljaoui, Y. A. Khan, W. Chammam, S. Z. Abbas

**Affiliations:** 1 College of Information Science and Engineering, Shandong Agricultural University, Tai’an, China; 2 Department of Mathematics, Huzhou University, Huzhou, 313000 People’s Republic of China; 3 Hunan Provincial Key Laboratory of Mathematical Modeling and Analysis in Engineering, University of Science & Technology, Changsha, 410114 People’s Republic of China; 4 Department of Computer Science and Information, College of Science at Zulfi, Majmaah University, P.O. Box 66, Al-Majmaah, 11952 Saudi Arabia; 5 Department of Mathematics and Statistics, Hazara University Mansehra, Dhodial, Pakistan; 6 Department of Mathematics, College of Science Al-Zulfi, Majmaah University, P.O. Box 66, Al-Majmaah, 11952 Saudi Arabia

**Keywords:** Feature selection, Health care, Hybrid classification, Human-physic, Retrieval-ranking, Prediction, Machine learning

## Abstract

**Background and objectives:**

The ideal treatment of illnesses is the interest of every era. Data innovation in medical care has become extremely quick to analyze diverse diseases from the most recent twenty years. In such a finding, past and current information assume an essential job is utilizing and information mining strategies. We are inadequate in diagnosing the enthusiastic mental unsettling influence precisely in the beginning phases. In this manner, the underlying conclusion of misery expressively positions an extraordinary clinical and Scientific research issue. This work is dedicated to tackling the same issue utilizing the AI strategy. Individuals’ dependence on passionate stages has been successfully characterized into various gatherings in the data innovation climate.

**Methods:**

A notable AI multi-include cross breed classifier is utilized to execute half and half order by having the passionate incitement as pessimistic or positive individuals. A troupe learning calculation helps to pick the more appropriate highlights from the accessible classes feeling information on online media to improve order. We split the Dataset into preparing and testing sets for the best proactive model.

**Results:**

The execution assessment is applied to check the proposed framework through measurements of execution assessment. This exploration is done on the Class Labels MovieLens dataset. The exploratory outcomes show that the used group technique gives ideal order execution by picking the highlights’ greatest separation. The supposed results demonstrated the projected framework’s distinction, which originates from the picking-related highlights chosen by the incorporated learning calculation.

**Conclusion:**

The proposed approach is utilized to precisely and successfully analyze the downturn in its beginning phase. It will assist in the recovery and action of discouraged individuals. We presume that the future strategy’s utilization is exceptionally appropriate in all data innovation-based E-medical services for discouraging incitement.

## Introduction

In the present life, online web-based media is especially moving among individuals where media substance is being public and placement. These mutual things/items are seen and enjoyed by different clients on online media. This allocation and loving are adding to the ubiquity of the thing/item. For instance, a sentimental film of a renowned entertainer, whenever labeled, at that point, different clients looking through sentimental motion pictures of his No.1 entertainer can go without much stretch access from this labeling. Simultaneously, business gatherings or suppliers routinely dissect such online media to recover their item excellence. For instance, a film of a specific entertainer getting audits, for example, the film’s content is acceptable, yet the nature of video needs to improve. Clients may likewise select likings and rating symbols for movies or items. For the most part, these online media keep restricted data like clients, things, and clients’ criticism. The main factor of this data is clients who assume an essential part in such media. Especially the online-media planned distinctly for the clients devoured online substance and gave criticism to the things. To get real criticism, clients likewise keep their essential data, for example, name, interest, birthdate, and so on, the media. The following significant media piece can be client-created content like writings, photographs, recordings, and any item like camera, film, PC, and so on internet advertising. The other piece of the online-media criticism was gotten by the clients, which could be of some extent. Such inputs could be in varieties relying upon the utilization of the item. To decrease the manuscript content, the online media likewise present labels/marks to the clients for criticism. Such labels create simply for the clients to provide criticism.

Exact and successful information removal from the content is awkward because of unstructured content data. In this way, analysts are inclined toward the input through labeling; enjoying and naming makes extraction data from the crude information exceptionally simple. A novel technique has planned to break down the labels and appraisals of online media for clients just as business parties [1]. These investigations inform how specific clients allot evaluations and labels and concentrate designs. With this investigation, it is seen that all inputs for motion pictures of war are given simply by guy commentators in New York. Creators tackled such issues by utilizing slope hiking and heuristics and hierarchal agglomerative bunching techniques depicted in [1]. Later in [2], the authors characterized tweeter content as a great wellspring of information forecast.

Fame expectation is one of the most significant pieces of straight-out examination. A strategy in [3] has been proposed to identify the endorsement of the film set up as per movement on the online media like Netflix, Hotstar, Twitter, and so forth in another writing [4], the produced traffic for the certain tape on YouTube has been anticipated during connected substance accessible on Twitter. Vashishtha and Susan [5] consider sentiment analysis (SA) for human emotion evaluation. Following comparative methodology [6], a technique has already been proposed to foresee the film notoriety dependent on online web-based media input utilizing manuscript removal. In [1], creators have known a system to deliver the important realities from the 4500+ remarks of normal appraisals 8.5 about cell phones. For instance, male analysts under 30 from Delhi might want to purchase a specific cell phone, while young people would want to purchase an excessive cell phone with a great camera.

By and by, the study is expanding in ML-driven purposes, incorporate robot revelation [7–9], autonomous automobiles [10], sibilance protection [11, 12]. Application-level semantics of web-based video resources have been getting pervasive in a massive range of utilizations. Pictures [13], recordings, and sound are enormous wellsprings of information, from which more data and settings can be accepted.

As we have seen from the above examination, human feelings are related to audits communicated on online web-based media. These feelings may show diverse conduct of individuals like upbeat, pity, uneasiness, and upset psyche. For instance, an upbeat individual will get a kick out of the chance to see interesting recordings on online web-based media and give positive audits. In contrast, a dismal individual will jump at the opportunity to post some off-kilter audits to show his steamed brain. One of the genuine sicknesses is misery, which is a psychological problem. The indications like the sentiment of tension, pity, sporadic rest, and upset psyche are found in discouraged individuals. Loss of energy and interest, focus issues are likewise some different manifestations available in such conditions. The seriousness of a downturn can cause self-destructive endeavors [14, 15].

The remaining of this research is organized accordingly as Section 2 provides a detailed overview of the literature, Section 3 signifies relevant technical background of proposed multi-feature hybrid classification. The application is subsequently demonstrated in Section 4, while Section 5 concludes this research with an outlook for future research.

## Brief literature review

Misery is seen by taking a gander at the individual how he acts, feels, notices, or thinks. Psychological well-being proficient necessities a framework that could analyze this significant issue at the beginning phase. This is because it originates from interior organic frameworks’ unsettling influence, an exceptionally intricate framework [16]. It isn’t anything but difficult to notice individuals’ temperament utilizing sensors in this way troublesome in recognizing misery. Analysts saw that underlying compromising signs are conceivable just in hazardous progress periods [17, 18]. In such conditions, it has represented a great test for both scientists and clinical specialists. Human feelings are related to surveys communicated on online web-based media for which analysts examined various manners.

The online web-based media exceptionally unpredictable organizations of clients. Facebook and Twitter are well-known online web-based media where huge clients can compose situations departure on their psyches, divide recordings, and be similar to them. The connection thickness and notoriety of tweet and prominence and dissemination profundity explain unenthusiastic and optimistic relationships separately. The fame of things among double cross lines is additionally profoundly related. Future ubiquity of a thing is anticipated by taking a gander at its prominence in the past course of events and connection thickness of clients posted that thing. The well-known techniques are calculated relapse, which is utilized for fleeting investigation of tweets dependent on the tweet’s prominence in past timetables [19]. The investigator in [2] discovered ubiquity in the past schedule is straightforwardly associated with the last fame of thing if there should arise Digg and YouTube.

The notoriety of things on video distribution destinations incorporates Netflix, Digg, Youku, and so on follow rich get more extravagant impact if thing’s fame is for long. It is also discovered that things well known in past timetables are bound to be famous in future events [2]. Along these lines, prevalence will go for a long time. In [6], researchers have indicated that Youtube recordings’ fleeting notoriety is a decent indicator for its last ubiquity. The supply processing has given a novel technique, an enormous repetitive neural network (RNN) that regards composite non-linear wonders in the underlying ubiquity and the last prominence [20]. On YouTube, there two classes of recordings seen by clients have been noticed depending on their fleeting example: one that shows an unexpected explosion of prevalence and blurs missing and the extra one that illustrates extended haul ubiquity [14].

In [20], creators have abused progressive bunching dependent on the recordings’ time arrangement [21]. has thought about more profound attributes of things (Youtube recordings) prevalence and given a model that considers distinctive agent things as indicated by worldly fame, rather than picking just single delegate things for the entire interval. 5 Worldly pattern forecast, for example, Twitter pestilence and irresistible infection pandemic. Since irresistible sickness and Twitter posts spread additionally observe same law, for example, spatial and worldly property as a substance on interpersonal organization, for example, degree dispersion [16, 22], bunch coefficient [23, 24], and network construction [25, 26] By allowing for the organization configuration highlights of the microbe irresistible infection spread can likewise be redacted same as an informal community [27]. These organizations can be said as a contact network [28]. It demonstrates the example of a connection that can source to the correspondence of communicable debilitated. In correspondence complex, every person or area is spoken to with vertices or hub of the diagram, and associates between individuals of the area are spoken to utilizing limits. Early on efforts, the authors in [29, 30] displayed the edges among individuals in a medical clinic or a city for respiratory illness broadcast [31]. has displayed the bipartite chart among the parental figures and patients in an emergency clinic. The main irresistible infection begins from any hub in write to system same as a Twitter post by any client, at that point it banquet to its neighbors. Without the scale model, an expected result is that the most established hubs consistently have the most significant number of edges [32] outlined the agglutination of prosodic and supernatural features from a group of sensibly selected features to appreciate hybrid audio features for enlightening the task of emotion recognition. In [33] investigator developed fuzzy entropy to tap sentiment quotients of online movie reviews and implemented the approach on shortlisting of words that help in sentiment cognition with the help of a combination of clustering, sentiment lexicon SentiWordNet, and fuzzy entropy.

The insightful model uses early ubiquity as the base diagnostic variable. Various endeavors have been done which incorporate different factors also. Qualities of substance maker, at times maker of the substance, assume a significant part for settling on choice while making expectations, such as the news from a notable distributor, thing from the understanding brand, melody from the notable artist, and so forth prevalence of the substance. Literary highlights, certain words or key expressions, or key expressions that are notable to the purchasers also assume a significant job. Like in the hour of US shaft, the news and sites that incorporate content identifying with posts will get more consideration. Content classification of the substance additionally plays benefits for its fame; for example, news from legislative issues would be perused by more populace like a cell phone is a more worthy class than any lively pack. Social sharing review conduct, client’s activity during sharing can be utilized to anticipate the ubiquity. For example, Yippee Zinc, that permits the client to control video content progressively.

In all actuality, numerous organizations, such as the web, can increase the most extreme number of edges in a brief timeframe. In [34], the investigator developed an emotion recognition system with the help of a deep learning approach for emotional Data and evaluated the audio-visual emotional databases’ performance. In [35], the author developed efficient multiple features sentiment classification algorithm with SVM and Fuzzy Logic for online text reviews in social circles. Investigator in [35] validated the developed model on *the Twitter data set and utilized the model for prediction purposes.* In [31], the investigator found that continuously networks show serious conduct, that a few hubs may draw edges from different hubs. Therefore, they proposed a summed up particular connection model, in which a youthful hub with a couple of edges can gain numerous edges at a high rate based on a wellness boundary. This boundary offers the ability to hubs for rival other hubs for edges. This investigation shows that human feelings are a lot associated with audits communicated on online web-based media.

In this examination, the proposed framework is utilized to analyze the downturn precisely in its beginning phase. It helps in the recovery and treatment of discouraged individuals. We recommended that utilizing the proposed technique is exceptionally solid in all parts of E-medical care for pushing down incitement.

## Method and material

### Method

#### Optimal characteristic assortment

We present a two-phase underline determination approach dependent on the RFE and EFS type of computation. The techniques for highlight guess from the EFS phase as the information and heuristics for the resulting RFE reducing point. In the first period, we utilize the EFS computation to get comprise many and choose important places of interest; in the following phase, the constituent assessment accomplished from the main phase is used to manage the beginning of the boundaries required for the hereditary calculation. The coordinated-based internet searcher has been applied to discover agreeable redacts. The EFS FS subsystem contained of three significant modules:

1. Data discretization
2. Feature extraction utilizing the calculation, and
3. Feature decrease utilizing the heuristic RFE decrease calculation we created

#### Hybrid classification

**Fig. 1:**
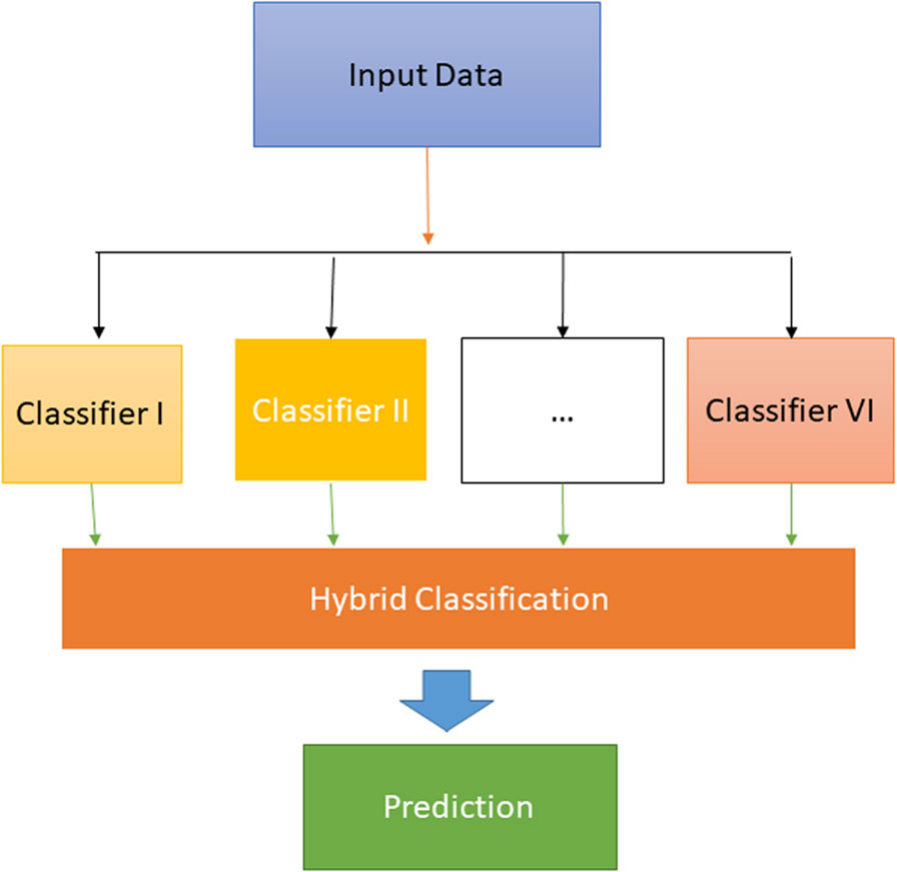
Hybrid Machine Learning Classifier

The machine learning pipeline approach of AI (ML) incorporates various stages for preparing the model. In any case, the term pipeline is misdirecting as it suggests a one-bearing correspondence of information. ML pipelines Fig. 1 are repetitive and iterative as each progression is rehashed to consistently expand the precision of the model and acquired a fruitful calculation. To make reasonable ML models and get the most incentive from them, available, versatile, and strong capacity arrangements are basic, making them ready for on-premises object stockpiling.

A half and half characterization calculation is created utilizing a group learning strategy. There are various outfit education methods. In this form, we have anticipated the consequences of primary group learning strategies. The test consequences exhibited that the technique was amazingly performed. The general own of the crossbreed strategy is given in the segment of analysis.

Number prescient models are prepared to get familiar with the model utilizing the preparation dataset. Form the 1st point learn classifiers as

1 $$ D=\left\{\begin{array}{c}{x}_n, Data\  Set\\ {}{L}_{T,}\  First\ Learning\\ {}L, Second\ Learning.\end{array}\ \right. $$

The second stage of learning then integrates the individual learning models.

models the hybrid models are given as

2 $$ {h}_t=\left\{\begin{array}{c}{L}_t(D),t=1\to T,\\ {}{h}^t, Training\ Level.\end{array}\right. $$

Generate a new data set as

3 $$ {Z}_{it}=\left\{\begin{array}{c}\ {D}^T=\Phi, data\  set\\ {}{h}^t\left\{x(i)\right\},t\to n.\end{array}\right. $$

Train the second-level learner given below

4 $$ {D}^{\prime }=\left\{\begin{array}{c}D\left[U{Z}_i,y(i)\right],\\ {}{h}^t, where\ L\left({D}^{\prime}\right).\end{array}\right. $$

And compute the accuracy of the trained hybrid model as

5 $$ H(x)={h}^{\prime}\left\{T(x)\right\}. $$

Framework factorizations move toward can be functional to explain tweet pestilence and infection pandemic expectation [26]. has given a strategy dependent on the lattice decay technique for anticipating occasional sickness by utilizing irresistible gastroenteritis brought about by Noro infection in Japan. We can display the concealed highlights, for example, individual tainted length, infection point, geographic, and auxiliary highlights of the phone organization. Feelings set off by web content are exceptionally related to its notoriety. The subjectivity of the verbal communication for the news has assumed a function for foreseeing re-tweets. Languages communicated in change by clients likewise influence the new clients. A little distinction among Twitter extend expectations, and sickness increase is carefully following the contact network’s auxiliary property just as the level of infection [28]. Given a probabilistic model, each edge from a tainted hub in an e-mail organization will move to the highest point after *t* time.

Consider the phase of a separate time. If summit *i* is infectious for *τ* time phases, next to the chance that *j*i will contaminate j is *T*_*ij*_ is given in Eq. 6, which can be easily understood by algorithm-I given below

6 $$ {T}_{ij}=1-\left(1-{r}_{ij}\right)\tau $$

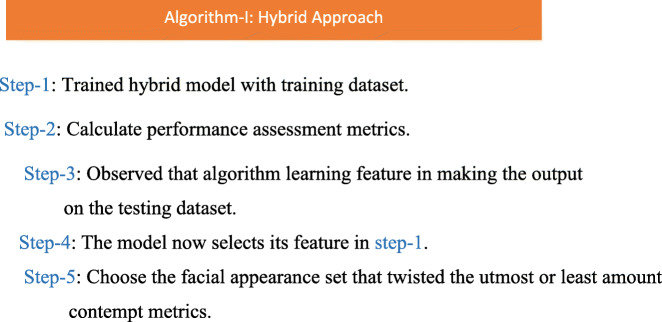

#### Proposed method evaluation metrics and assembly learning algorithm

A confusion matrix (CM) having data about genuine and anticipated orders performed by a classifier. The presentation of such a strategy is commonly assessed applied to the information in the network. In the CM, TP is the number of genuine positives. FN is the number of bogus negatives. TN is the number of genuine negatives at last; FP is the number of bogus positives [2]. The exhibition’s assessment measurements are communicated in Eqns.7–12 Exactness: Precision is numerically expressed as

7 $$ {A}_{cc}=\frac{TN+ TP}{TP+ TN+ FP+ FN}\times 100. $$

Sensitivity/Recall is mathematically expressed as.

Sensitivity (S_n_) /Recall:

8 $$ {S}_n=\frac{TP}{TP+ FN}\times 100. $$

Specificity is mathematically expressed, as shown in Eq. 9. Whereas precision is given in Eq. 10

9 $$ {S}_n=\frac{TN}{TN+ FP}\times 100, $$

10 $$ Precision=\frac{TP}{TP+ FP}\times 100. $$

F1- score: F1-score is mathematically expressed as

11 $$ F1- score=2\frac{Precision\times recall}{Precision+ recall}\times 100. $$

MCC: MCC is mathematically expressed as; which is represented by

12 $$ MCC=\frac{TP\times TN- FP\times FN}{\sqrt{A_1+{A}_2+{A}_3+{A}_4}}\times 100. $$

Where, *A*_1_ = (*TP* + *FP*), *A*_2_ = (*TP* + *FN*), *A*_3_ = (*TN* + *FP*), and *A*_4_ = (*TN* + *FN*).

ROC-AUC: The ROC bend is used for deciphering the forecast execution of the classifier. It is generally plotted to utilize the genuine positive rate versus the false optimistic speed, as the visionary model’s separation boundary is different. The territory under the ROC bend (AUC) is usually exploited and recognized in characterization, contemplating that it provides a high synopsis of a classifier’s execution.

**Fig. 2:**
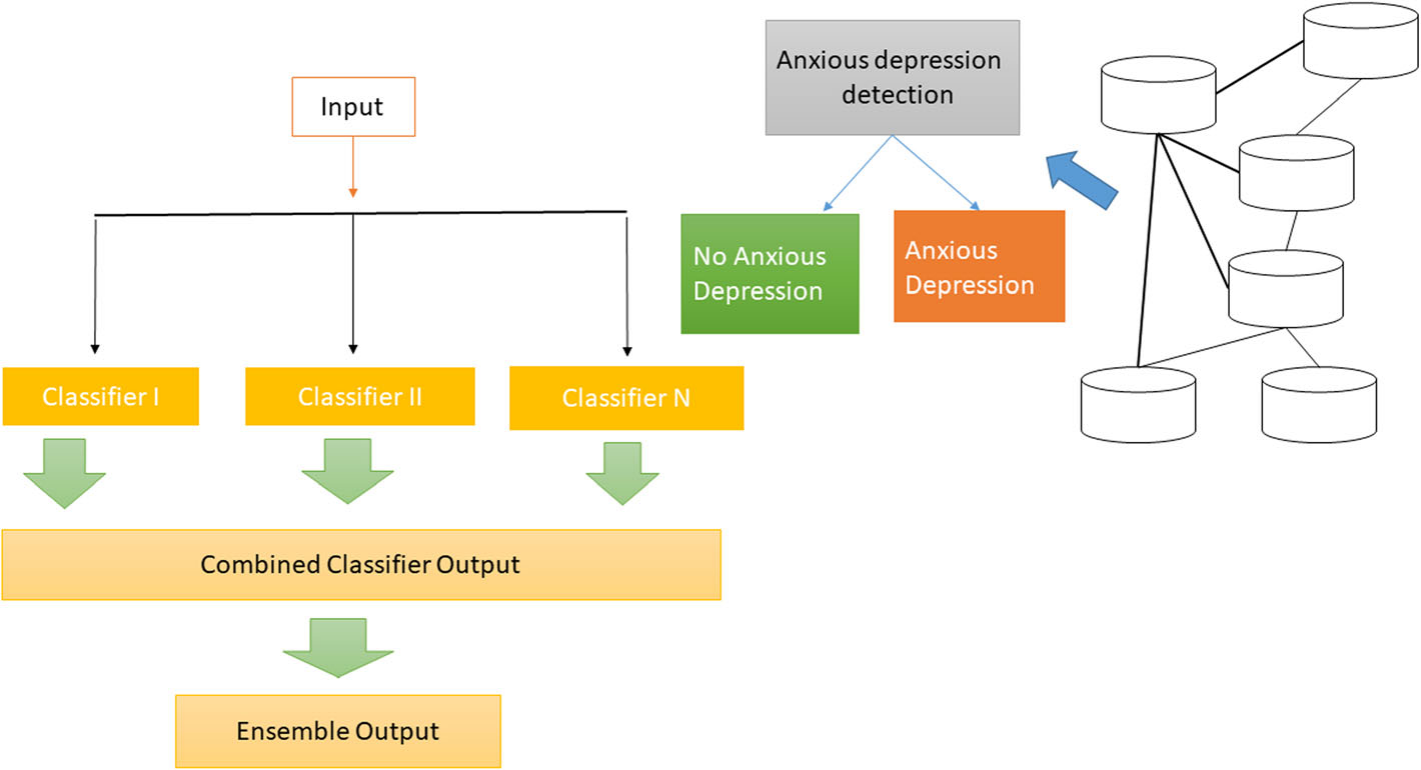
Hierarchy of ensemble process model of a human-emotion data set

In outfit education calculation yield of additional than one knowledge, calculations are consolidated to provide additional precise outcomes. To accomplish great gathering calculation, the classifiers are picked to not make any mistakes in various portions of the example space as introduced in Fig. 2.

Bagging [2] and Boosting [29] are two exceptionally famous gathering strategies. These techniques use Re-sampling strategies before learning by various classifiers. Packing and Boosting are extremely viable with choice trees. If the classifiers make a mistake in a similar example space, then sacking and boosting won’t be more successful. Henceforth difference is required between classifiers. This is suspicion that if the classifier’s mistake rate is fewer than 50% and classifiers create blunder in various spaces or can’t help contradicting one another, at that point, by brushing limitless classifiers, we can diminish the mistake to zero.

##### Planned projecting structure for forecast of affecting physic

The pseudo-code of the projected method for affecting physic prediction is given in algorithm- II. The proposed method flow chart system is given in Fig. 4 below. 
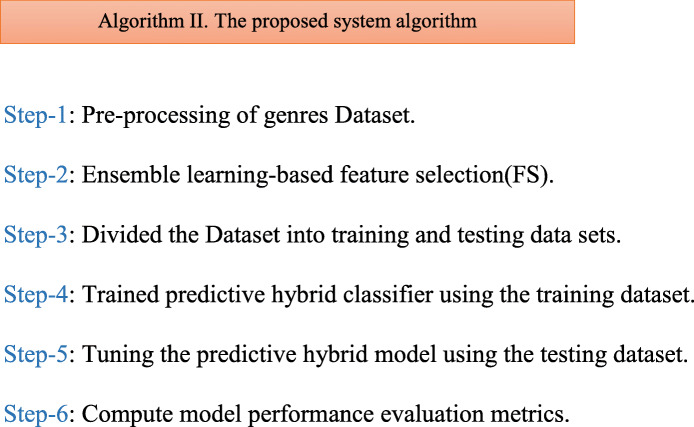


### Materials

#### The data

The Dataset utilized in this exploration is taken from MovieLens Types Tag-Rating information base, which is online accessible at https://grouplens.org/datasets/movielens/most recent/. An overall-based amusement business organization gives the MovieLens Classifications Tag-Rating Dataset at their official site and online on the UCI information base [29]. The Dataset has three sub-record that is interface subset, tag-subset, sorts subset. There is a size of information that contains 7533 motion pictures, 864,581 labeling, and 5000 clients. Five thousand subjects alongside 32 characteristics and 30 highlight genuine qualities. For all the information of the Film focal point, the time is considered day.

**Fig. 3:**
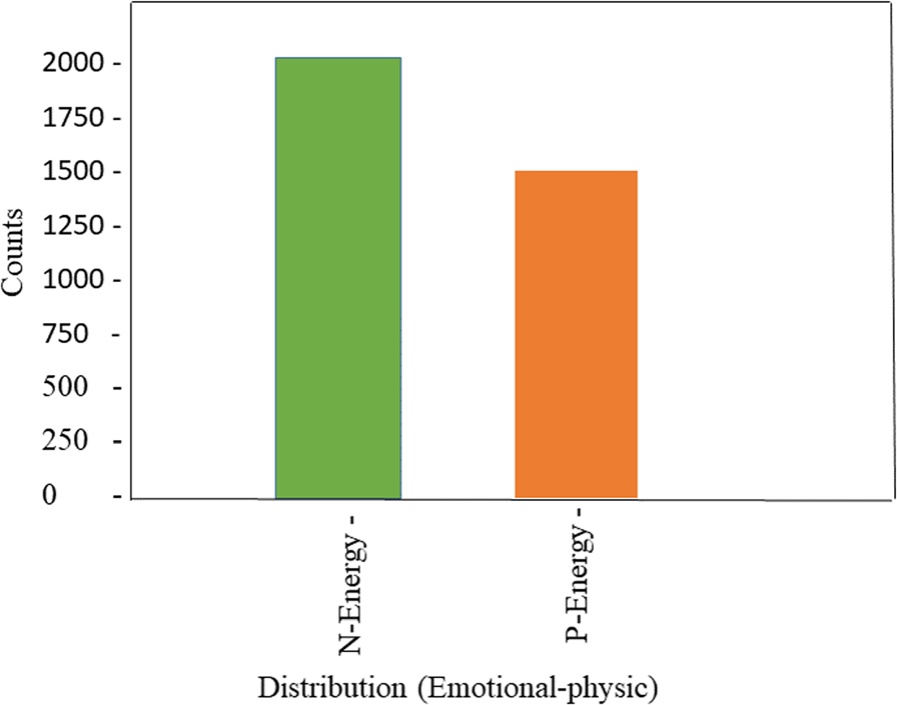
The (depress and healthy) people ratio of emotional-physic in the Dataset

Moreover, in this examination, the Dataset has been parted into 75 and 25% separately for preparing and testing of the model. Moreover, to check the model exhibition, various measurements are figured consequently. The exploratory outcomes, arrangement, and designs are produced for the visual introduction. The total of what recreations have been acted in the R-studio programming climate is easy to understand and openly accessible online on an Intel(R) Center – i5 - 2400CPU@3:10GH, Smash 3 GB, and Windows 10. The proportion of enthusiastic physic (push down and sound) individuals in the Dataset are introduced in Fig. 3 below.

### The contributions of this are as follows

1. This study gives significant exact perceptions of the picked long-range interpersonal communication, which contains enormous information about the client and client’s collaborations with their passionate physic.
2. We concocted a model to foresee the client’s passionate mental conduct and affect others’ proportion about things or motion pictures on long-range informal communication.
3. We have considered the dataset MovieLens, types, and labeling cooperation for affirming our model’s precision.

## Empirical result and discussion

**Fig. 4:**
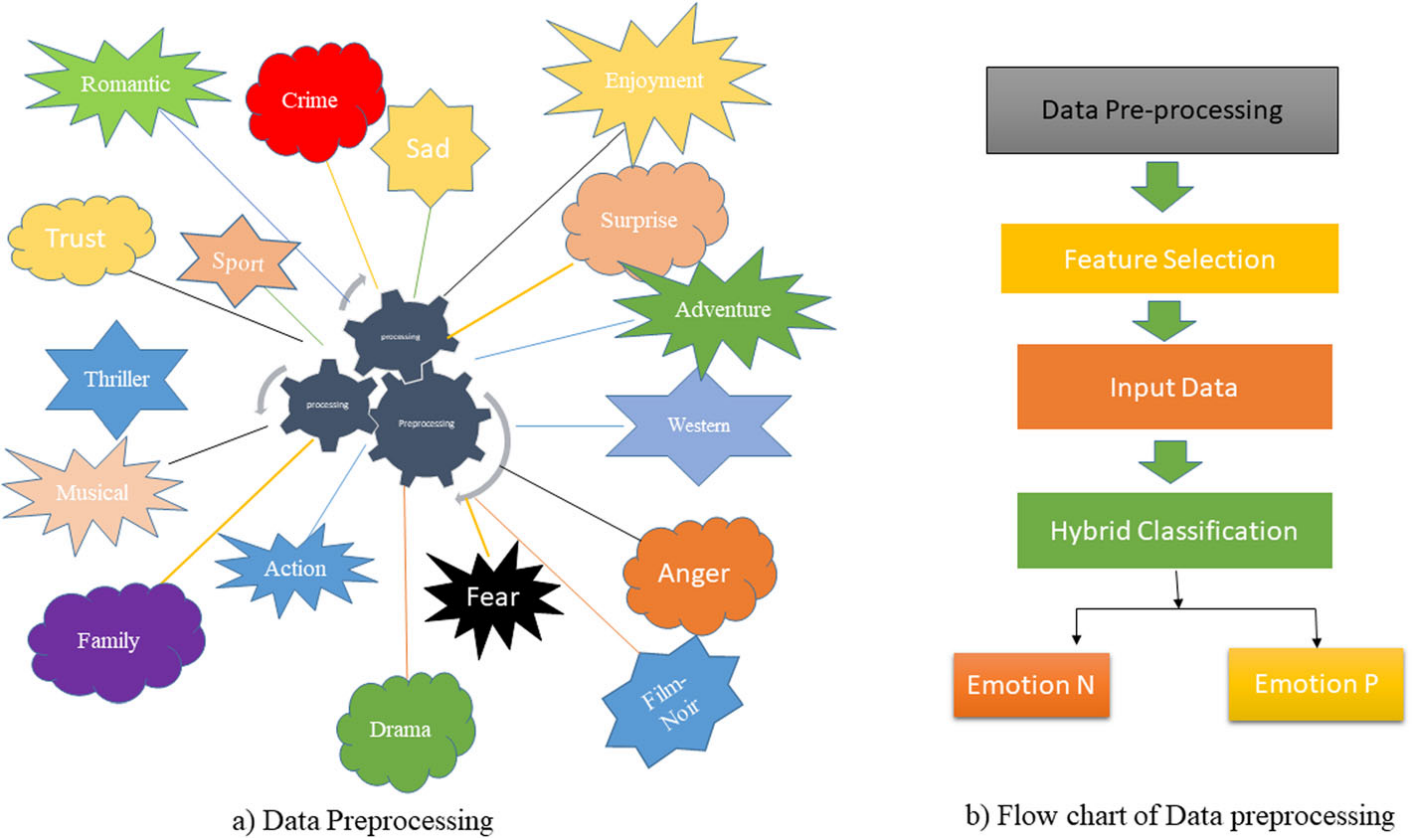
Data Preprocessing Model and Flow Chart of Human-Emotion

The proposed half and half order strategy have two sections:

1. Feature Selection (FS) applied the Recursive Element Elimination (RFE) subsystem and
2. Data arrangement utilizing the Mixture grouping framework. A stream graph of the proposed technique is given in Fig. 4. This segment presents kinds of feeling data investigation on informal communities, relative correlations of procedures, and their outcomes are talked about in subtleties in the coming sub-areas. In this part,

We played out the analyses for passionate types of physic expectations utilizing Group Learning Calculation for reasonable FS. The ML prescient Half and half model has been utilized for the expectation of melancholy physic.

Information bases of passionate physic accompany two confounded issues;

1. Data in the clinical division is regularly secured and complex to get to, making it difficult to analyze results among dissimilar techniques.
2. Information generally encloses a modest quantity of positive models; however, significantly more negative ones (scenes are typically not the standard). It’s a lot simpler to gather ordinary information contrasted with pertinent cases).

Another strategy for unbiased identifying wretchedness could be increased pulse [26] and voice accounts [28]. Nonetheless, these methods have not been concentrated in wretchedness, most likely because the assortment of such information is an unquestionably more intricate and testing position than utilizing a basic wrist-worn act graph to collect engine action information [27]. have proposed socio-designs irresistible information based plan, where principal social brain science is considered about each item whose classifications is in the type of feeling. Which is accessible in conduct or in the cerebral cycle of each livelihood animal in the world? In any case, this energy relies upon the state of mind of the client, which takes it or passes it to others in their situation as harmful as sorrow or in as an improving feeling of different as a good feeling.

To examine the pointer’s accuracy and intrigue various statistics, we utilize Kinds of Labels MovieLens informational compilations. MovieLens informational directories enclose film categorizations, assessments, stickers, and online media enormous Dataset includes clients’ separator post relations.

**Fig. 5:**
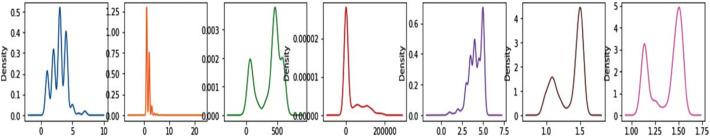
Frequency distribution of the human-emotion Dataset

This is the sequence that contains like and classification purposes with time beats. A little task finishes on surveillance film. Our model has selected slight separation from every by randomly different clients – who have appraised not many (3–10) films. The first evaluation was as arithmetical, we have measured the association among the customer and item which editorial have gotten senior to two assessments and incorporated kinds numerous. The frequency distribution of the human-emotion Dataset is shown in Fig. 5.

**Fig. 6:**
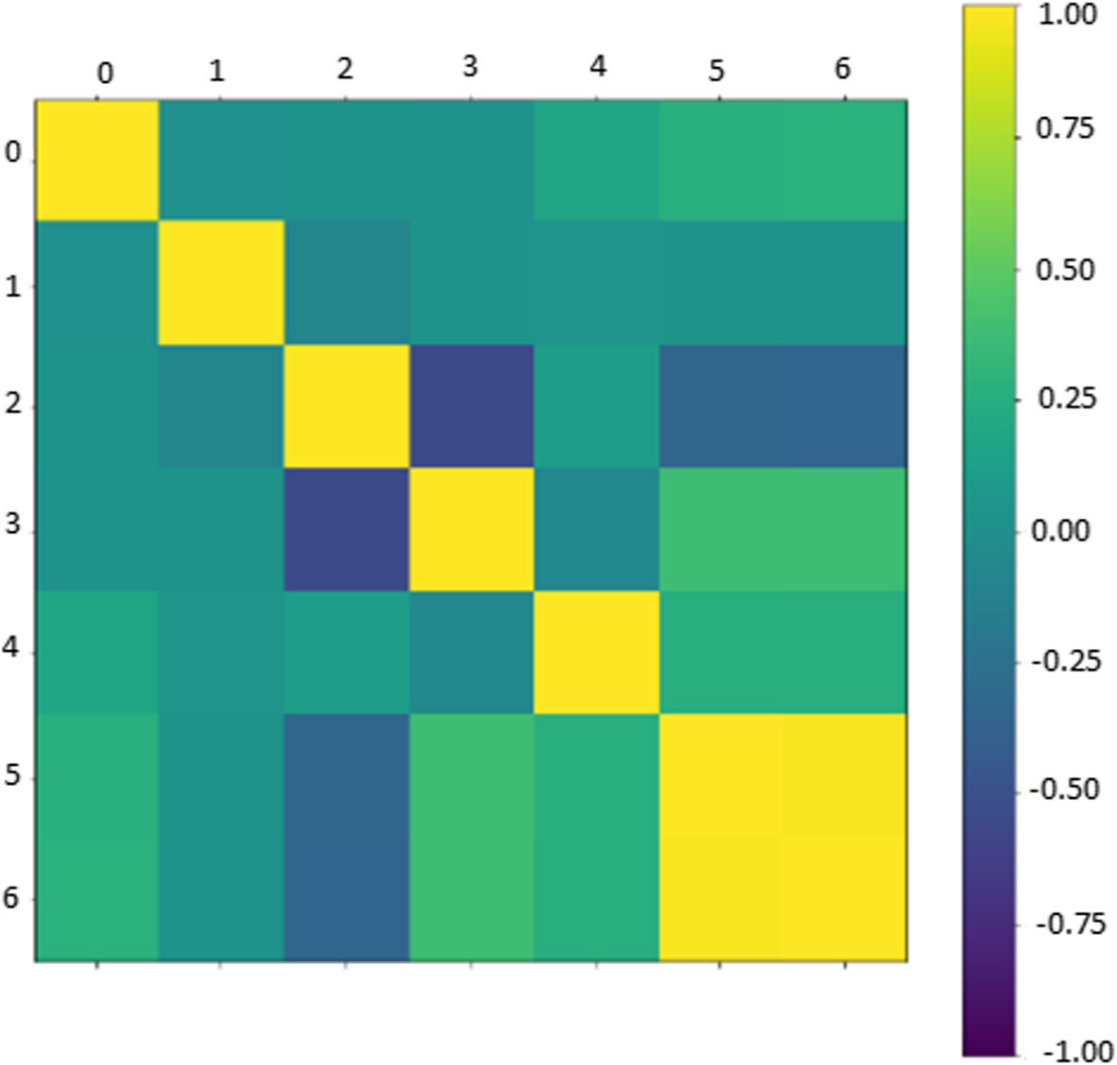
Correlation matrix indicating the relationship between users and posted on the wall

MovieLens in sequence encloses 7533 motion pictures, 864,581 labeling, and 5000 clients where their types are available 670. On the occasion that customer has placed on a partition, there will be a relationship between the client and the divider; self-impacted is removing by reducing the client’s association and its divider post. For all the information on Film focal point, the time is considered day. Correlation matrix indicating the link between users and posted on the wall see Fig. 6.

### Data pre-processing

The information pre-prepared found the running season of display and improve the classifier execution. The preprocessing methods, which incorporate omitted worth erasure, standard scalar, and min-max scalar, are generally used in the Dataset preprocessing. Standard Scalar (SS) that each element has signifies 0 and distinction 1; accordingly, all highlights have the equivalent coefficient in equivalent reach like [0 1]. Min-Max scalar moves the information in such a strategy that all highlights are run in 0 and 1. The highlights that have no incentive in the line are erased from the information base.

#### Preprocessing dataset results

**Fig. 7:**
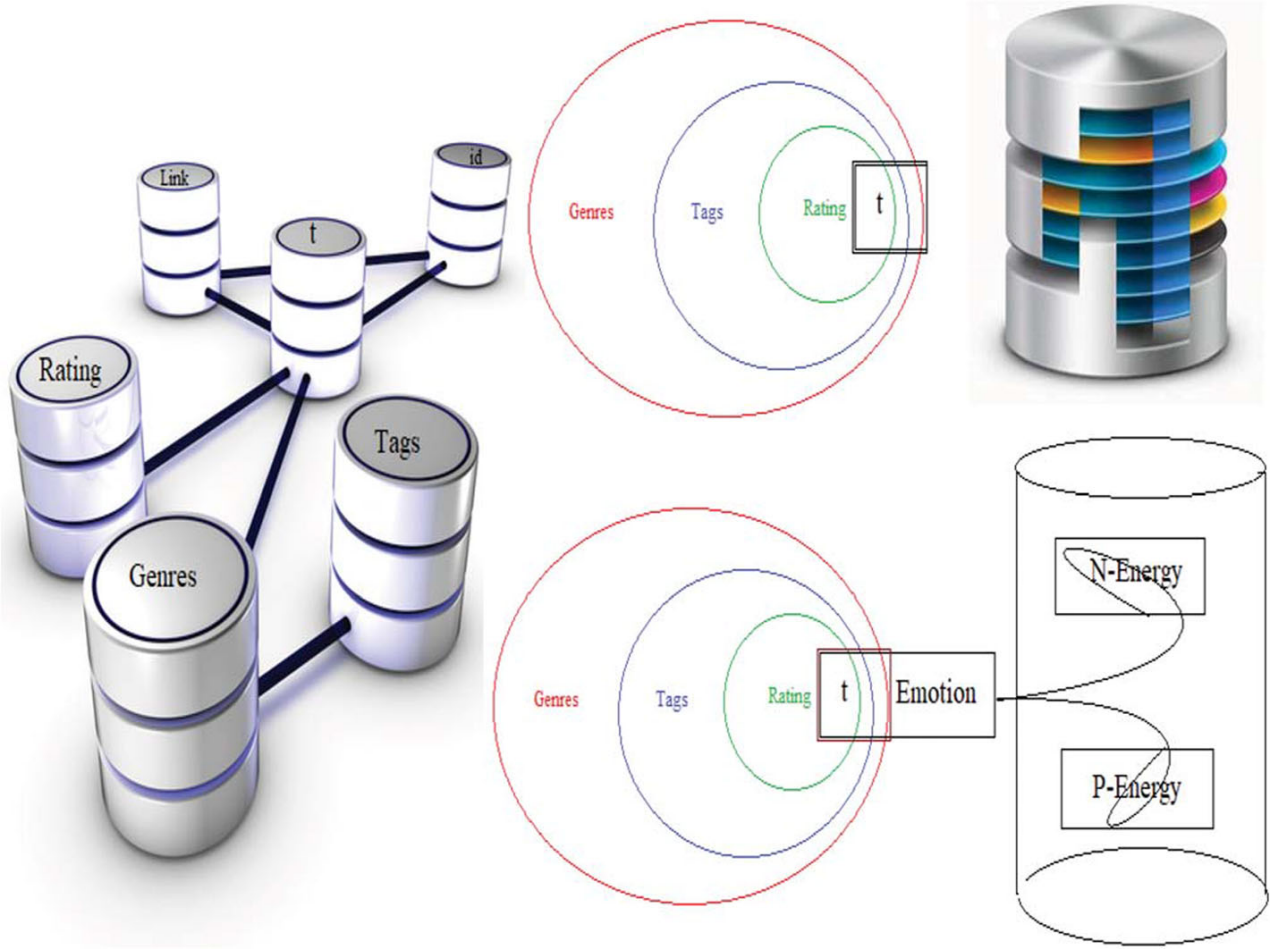
Hierarchy of Pre-processing of Input Data of human-emotion

The Dataset has 3569 examples with 12 ascribes, which are introduced in Table.1 alongside not many measurable activities, which are determined naturally. The class appropriation depends on negative and positive physic in the type of enthusiastic energy as subjects in a dataset introduced in Fig. 7 above.

**Table 1:** Decomposition of human-emotion Dataset into different component

	0	1	2	3	4	5
0	−2.08027e-09	− 2.18993e-06	− 0.000225908	−0.979433	0.2005	0.0226171
1	−1.10172e-09	−1.96008e-06	−0.00019204	−0.0802035	− 0.489725	0.86818
2	−3.17951e-07	−0.00174933	−0.999998	0.00047263	0.00113015	0.000459961
3	−2.32245e-05	0.999998	−0.00174934	−2.54652e-06	−3.4531e-06	−3.12346e-07
4	−3.04051e-09	−5.78577e-06	−0.00127446	−0.185145	− 0.848509	−0.495734
5	−1	−2.32239e-05	3.58583e-07	2.59765e-09	2.42321e-09	3.6474e-10

### Replication results

This segment covers up new situation mechanism knowledge for the mixture organization imitation results where characteristic input considers during the company knowledge algorithm.

#### Algorithm collection and results of HC spot dataset

Learning Highlights as opposed to using the Dataset’s entirety, FS calculations are applied for this reason. The yield of more than one learning calculation is joined to accomplish great group classifiers box dodging mistakes in various parts. These strategies use Re-testing procedures before learning by various classifiers. Suppose the classifier’s mistake rate is less than half percent, and classifiers make the blunder in various spaces or can’t help contradict one another. In that case, we decrease the mistake to zero at that point by crossover classifiers.

The ML pipeline results as an HC model estimated to recognize the emotional human-physic on the fundamental feature-set. The numerous designated feature subsets produced by the learning algorithm are listed in Table 2. A respective nearest neighbor also mentions in Table 3.

**Table 2:** Non-Scaled Spot Hybrid Classification Outcome of human-emotion data set

Value I α	Value II β	Nearest neighbors γ	Statistical.Sign ***P***-Value
LR	0:670071	0:031201	0.145
LDA	0:695223	0:023165	0.631
KNN	0:777380	0:031219	0.451
CART	**0:875027**	0:022399	**0.011**
NB	0:684118	0:026654	0.231
SVM	0.881639	0.019073	0.152

**Table 3:** Tune Scaled Hybrid Classification Outcome of the human-emotion data set

Value I α	Value II β	Nearest neighbors γ	Statistical.Sign ***P***-Value
Scaled-**LR**	0.700392	0.031720	0.301
Scaled-**LDA**	0.695223	0.023165	0.225
Scaled-**KNN**	0.752228	0.023799	0.053
Scaled-**CART**	**0.881672**	0.024908	**0.009**
Scaled-**NB**	0.710005	0.039495	0.065
Scaled-**SVM**	0.741100	0.026663	0.0171

#### The comparison of algorithms

**Fig. 8:**
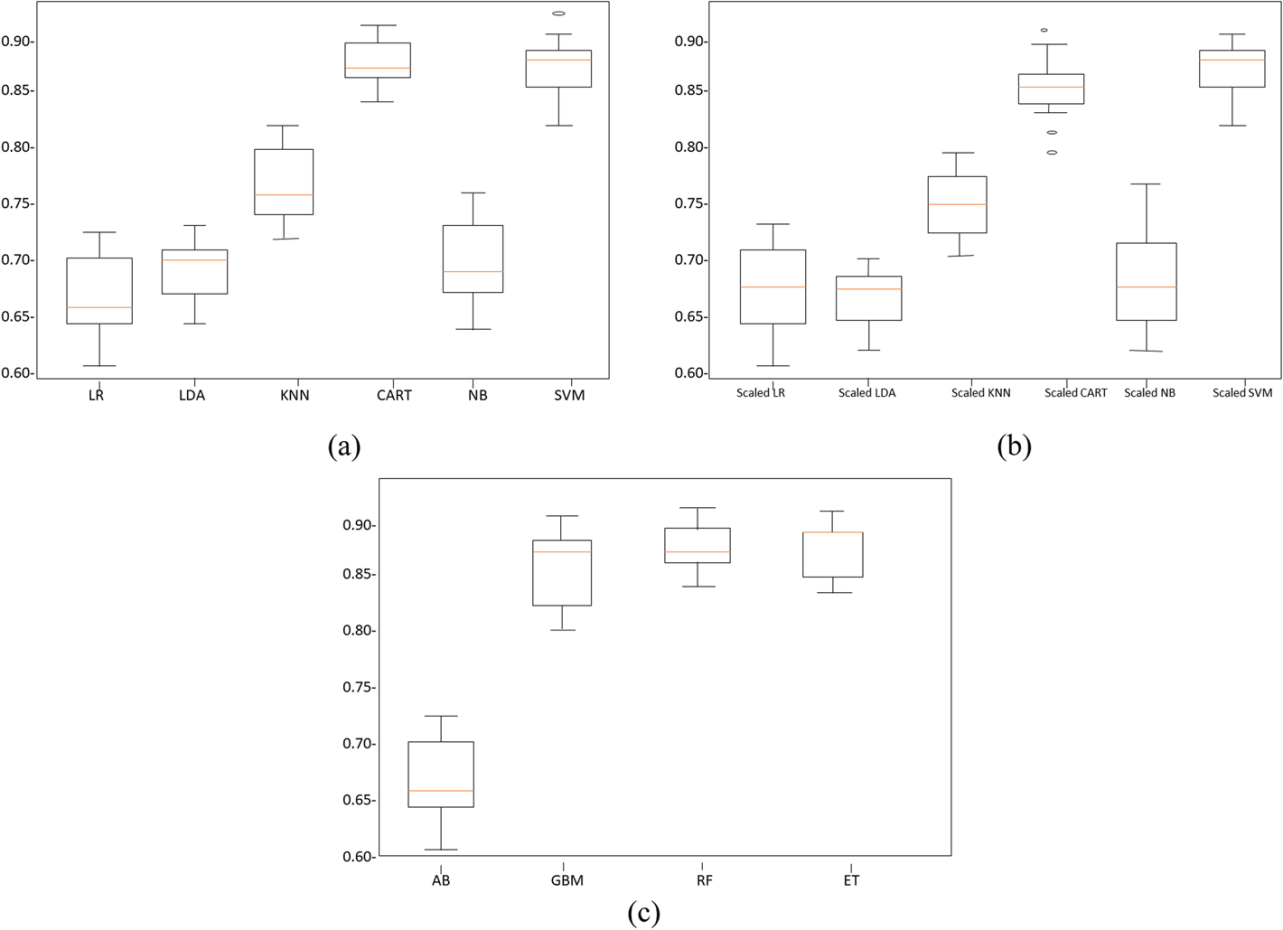
The Comparison of Algorithms: **a** Unscaled Human-Emotion Dataset (**b**) Scaled-Algorithms (**c**) Ensemble Algorithms

The comparison regarding the efficiency of Spot Check Algorithms is listed in Fig. 8(a), having a clear understanding.

The standard way of data distribution is the Box plots, about the outliers, data values are symmetrical or non-symmetrical, and the squeezes of data in grouped, the final shrewdness of data. Figure 8(a) illustrates the spot Check of the algorithm for the SVM, CART, KNN, LDA, NB and LR.

#### Standardized dataset HC results

Before use data to the classifier, it is necessary to conduct scaling of that data. A major objective of scaling data before processing is to remove features in greater numeric ranges. But before applying scaling on the given data, we need to ensure that we apply the same scaling method to the testing data before testing. Also, Scaling is a technique applied to normalize the range of independent variables of data. In processing data, it is also known as data normalization and is commonly performed during the data preprocessing step. On Standardize scaled Dataset, the modified results are listed through Table 3.

#### The comparison of scaled-algorithm and modified-scaled classifiers

Having the same reason as mentioned in the last comparison, Fig. 8(b) shown The spot check of the algorithm for The Scaled-SVM, Scaled-CART, Scaled-KNN, Scaled-LDA, Scaled- NB, and Scaled-LR.

It is perceived that AI simulations are determined through boundaries. These significantly influence the results of the learning measure. This boundary-tuning aims to trace the ideal-incentive for every boundary to advance the precision in the model. Natural streamlining of these boundaries will fetch the more efficient models.

Frequently in displaying, both boundary and hyper-boundary tuning are called for. What recognizes them is whether they precede (hyper-boundary) or after (boundary) a fit model. KNN is a generally basic order apparatus, yet it’s likewise profoundly successful a great part of the time. It gets bandied about that in roughly 33% of all gathering cases, and it’s the best categorizer. A third! This model might be little, however so too is it powerful. The Comparison of Scaled-Algorithms is shown in Fig. 8(b).

The majority’s vote decides the kind of Classification, and in case of a tie, the decision moves to the adjacent neighbor that is recorded first in training data. In the case of the two adjacent neighbors groups having matching distances with unlike names, the upshot will depend upon the information preparation request. KNN would have the option to recognize the three species from each other to shifting levels of progress, contingent upon what we set K as Table.4 is ideal: 0.782216 utilizing N-neighbors 1.

**Table 4:** Tune Scaled KNN Classifier Outcome human-emotion data set

N neighbors	Value I	Statistical.Sign P-Value	Value II	Statistical.Sign P-Value
1	0.78624	0.013	0.03300	0.001
3	0.76627	0.011	0.01836	0.004
5	0.75147	0.021	0.02262	0.0021
7	0.73150	0.013	0.02176	0.000
9	0.71449	0.031	0.02097	0.003
11	0.71301	0.027	0.02562	0.001
13	0.71671	0.017	0.03586	0.007
15	0.72189	0.025	0.02929	0.000
17	0.71893	0.022	0.02922	0.0015
19	0.72189	0.021	0.03677	0.000
21	0.73076	0.019	0.04111	0.000

The investigative classifier results are assessed for the discovery of passionate physic on the accessible list of capabilities and on different chose boundaries chosen by learning calculation. The classifier SVM boundaries esteem has been used in the entirety of our tests. SVM rbf prescient model exhibitions on a different joining of highlight subset have been classified into Table 4. Tuning boundaries esteem for ML calculations adequately improves the model presentation. There is a rundown of boundaries accessible with SVM. Here important boundaries that more effect on model execution, part, gamma, and C in Table 5 where best is 0.735207 utilizing C 1.7 with part ‘rbf’.

**Table 5:** Tune Scaled SVM Classifier Outcome of human-emotion data set

C	kernel	Case	Method-I	Statistical.Sign ***P***-Value
0.1		0.696746	0.033947	0.011
		0.707840	0.038778	0.009
		0.710059	0.036611	0.031
		0.669379	0.037225	0.025
0.3	Linear poly rbf sigmoid	0.696746	0.030322	0.000
		0.726331	0.035084	0.000
		0.737426	0.032385	0.000
		0.640533	0.028286	0.000
0.5	Linear poly rbf sigmoid	0.696746	0.030322	0.017
		0.727811	0.029701	0.000
		0.741864	0.029337	0.001
		0.611686	0.029381	0.001
0.7	Linear poly rbf sigmoid	0.696006	0.029449	0.007
		0.733728	0.030033	0.009
		0.741124	0.028504	0.000
		0.610947	0.027179	0.000
0.9	Linear poly rbf sigmoid	0.696006	0.028643	0.000
		0.731509	0.033354	0.013
		0.741864	0.027550	0.021
		0.608728	0.031313	0.000
1.0	Linear poly rbf sigmoid	0.699704	0.026675	0.013
		0.730030	0.030996	0.011
		0.732988	0.026132	0.000
		0.745562	0.030979	0.000
1.5	Linear poly rbf sigmoid	0.601331	0.029092	0.000
		0.699704	0.033734	0.007
		0.733728	0.032625	0.021
		0.745562	0.032338	0.040
1.7	Linear poly rbf sigmoid	0.598373	0.034895	0.025
		0.699704	0.026132	0.000
		0.735207	0.033558	0.031
		0.750000	0.031968	0.000

The portion boundary is tuned to take straight, poly, rbf and “sigmoid”. The gamma worth can be tuned by setting the boundary. The cost boundary tunes the C esteem. Tune scaled SVM with Piece ‘rbf’, with C esteem 1.7, give greatest 75% execution at generally speaking scaled Dataset in correlation with other portion like ‘direct’, ‘sigmoid’ or poly.

#### The ensembles-HC results and comparison

The HC is screened by ML pipeline, where the learning classifiers establish a set of algorithms and then, by a weighted vote of prediction, gives new data points. By the algorithms use, we achieve the error-correcting outcome as listed in Table 6. Where the noted outcomes as:

**Table 6:** Ensembles Classification result of the human-emotion data set

Value I α	Value II β	Nearest neighbors γ	Statistical.Sign P-Value
AdaBoost	0.676732	0.033831	0.025
Gradient Boosting	0.867582	0.034054	0.040
Random Forest	**0.882397**	0.033210	**0.001**
Extra Trees	0.862424	0.026097	0.031

AB = 67%, GBM = 86%, RF = 88% and ET = 86%.

Using the Boxplots with the same reasoning as in the previous algorithms, Fig. 8(c) show the spot check of the algorithm for AB, GBM, RF, and ET outcomes.

### A daily-life application of proposed model

**Fig. 9:**
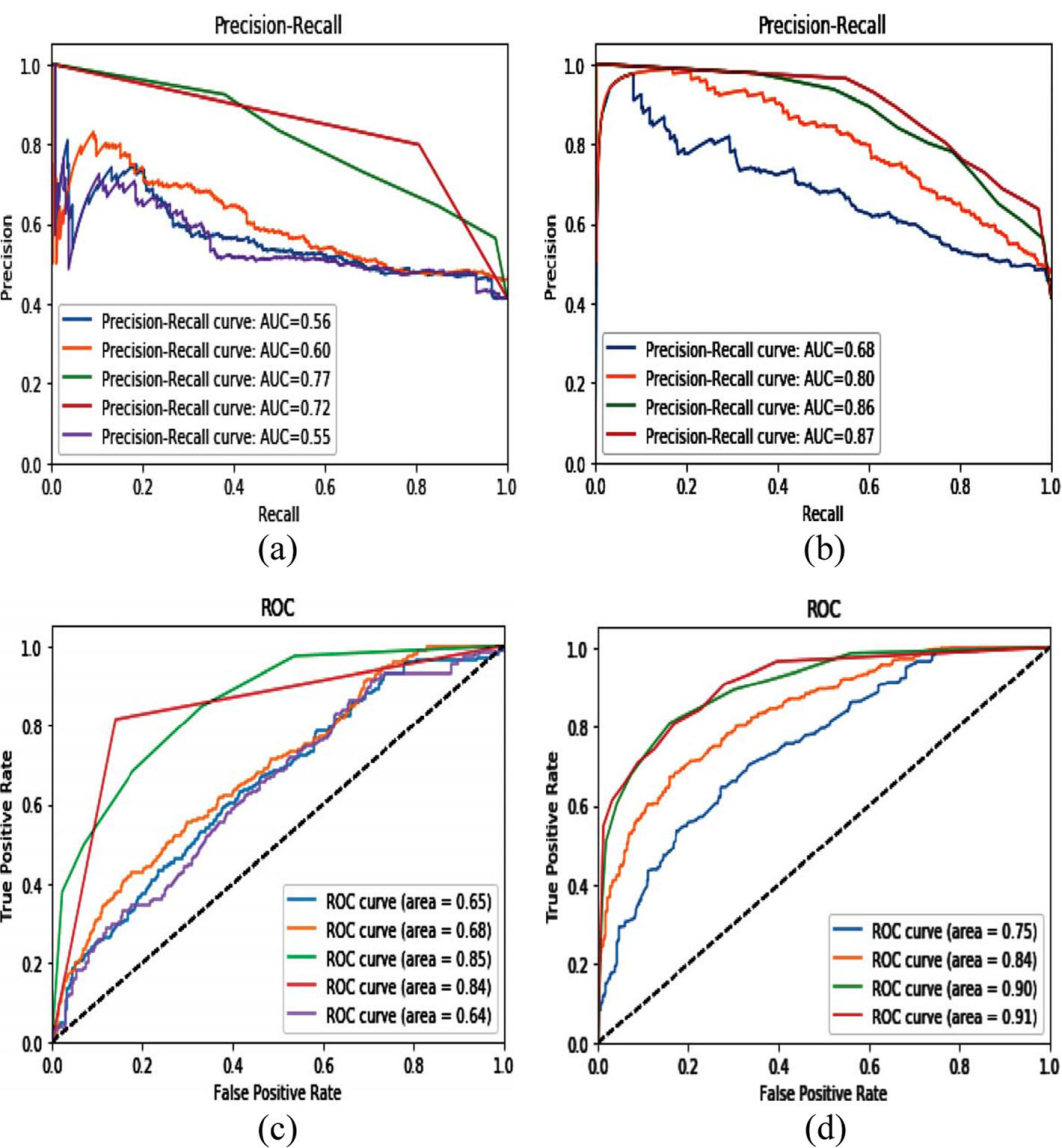
**a** Precision-Recall Cure for different value of AUC, (**b**) Precision-Recall Curve for Ensemble ROC, (**c**) ROC Curve of Human-Emotion Dataset, (**d**) Ensemble ROC Curve of Human-Emotion Dataset

Proposed arrangement model execution has been assessed for the discovery of despondency in the type of enthusiastic physic on the accessible list of capabilities and on various chosen mark subsets chosen by learning calculation. Proposed approach execution correlation is introduced in Fig. 9(a-d) underneath with order methods. The focused procedure’s presentation in terms of exactness is high as contrasted and the other way around.

In Table 3, the proposed strategy precision is contrasted and different methods. It present that the recommended approach increased high precision as contrasted and different conditions of the craftsmanship techniques. It is because of appropriate component determination by including choice calculation. That is why brain science said that human feeling is straightforwardly identified with things kinds, and the client continually looks through outer impetus to fulfill his interminable dream. Thus, labeling a great deal implies client spreading their present enthusiastic shelf circumstance in the type of the energy we considering as sure or negative relies upon our practices.

## Conclusion and future research

The passionate misery expectation method of web-things on the online-informal communities has been proposed in this examination study. A tale prescient model is grown, such things’ engaging quality that extras less alluring/appealing for an expanded term. It merits seeing that the model incorporates a huge advance with such a condition that web-content developments become dramatically throughout the time or stay direct for a brief timeframe. To assess the model for the necessary results, we used the genuine informational index and a particular connection-based model as a standard. Moreover, to remember a variety, we have considered the Dataset, e.g., on MovieLens, the enthusiastic advancement is quicker than content tight clamp online media. We have discovered that the presentation of the proposed model gets an extraordinary edge. Here in this study, we only considered MovieLens for a passionate substance like their sorts, labeling, and rating to foresee client state of mind. In the future one can consider more informational indexes and burrows the more patterns.

### Future investigation

Many research topics that one may expect in potential studies are brought up by exploring this research. We are going to address some of them here. i) One should apply the proposed method on electronic health care records and compare the efficiency. ii) Other possible future research directions will be to apply the proposed model and the deep learning approaches such as LSTM-RNN and Phased LSTM-RNN and compare the result in the presence of missing values. Finally, one may consider copula-based decision tree classification recently proposed by khan et al. [36] in the classification stage and compare the accuracy with the existing method. There are many other possible research points that are difficult to explain here, but one should think over it and work on it in the future.

### Limmitation of the study

To execute such a type of investigation, one required sufficient large data set contains a number of attributes for classification purposes having several levels or factors and are efficiently applicable in social networks, genetic, biotechnology, big data and online business etc.

## Data Availability

All results reported in this research were carried out in R-studio computational environment. Data used in this research is available online at https://grouplens.org/datasets/movielens/latest/.

## Abbreviations

AI: Artificial Intelligent
RNN: Recurrent Neural Network
IT: Information Technology
FS: Feature Selection
SN: Social Network
RFE: Recursive Feature Elimination
SS: Standard Scaler
SVM: Support Vector Machine
KNN: K-Neighbors Classifier
LDA: Linear Discriminant Analysis
LR: Logistic Regression.
HC: Hybrid Classification
